# Percutaneous Nephrolithotomy (PCNL): Standard Technique Versus Tubeless - 125 Procedures

**DOI:** 10.7759/cureus.4251

**Published:** 2019-03-14

**Authors:** Hamza Ichaoui, Ahmed Samet, Houssem Ben Hadjalouane, Amine Hermi, Houssem Hedhli, Mohamed Amine Bakir, Ramzi Khiari, Samir Ghozzi

**Affiliations:** 1 Department of Urology, Military Hospital of Instruction of Tunis, Tunis, TUN

**Keywords:** percutaneous nephrolithotomy, nephrostomy, tubeless, complications, hospitalization duration, postoperative pain, pcnl

## Abstract

Introduction: Tubeless percutaneous nephrolithotomy (PCNL) is the non-placement of a nephrostomy tube at the end of the procedure. The benefits of a nephrostomy tube placement are numerous as it provides adequate renal drainage. It may also tamponade bleeding and allow for an easier second-look nephroscopy. However, these advantages are mostly theoretical, and the majority of authors consider the nephrostomy tube as a source of morbidity.

Objectives: The aim of this report was to study the efficiency, safety, and morbidity of tubeless PCNL by comparing it to the standard technique.

Methods: This is a unicentric retrospective study of 125 patients who had undergone PCNL for renal lithiasis. We divided the patients into two groups: the standard PCNL group (n = 74) and the tubeless PCNL group (n = 51). The rates of good outcomes, complications, duration of hospitalization, and the degree of postoperative pain were compared between these two groups.

Results: There were no statistically significant differences between the two groups in age, gender, history, and the number of stones treated. There were more staghorn stones in the PCNL group with nephrostomy (p = 0.007) and more pelvicalyceal stones in the tubeless group (p = 0.037). Patients who had the standard PCNL had larger stones (p = 0.008). Patients who had a tubeless PCNL had more postoperative infectious complications than the standard PCNL group (p = 0.042). No statistically significant differences were noted for other complications, good outcomes (p = 0.13), postoperative pain (p = 0.51), and duration of hospitalization (p = 0.16).

Conclusion: According to the majority of authors, tubeless PCNL is considered a safe and efficient technique. It also provides advantages with less postoperative pain and duration of hospitalization. We believe that a selection bias may exist in most published work concerning routine nephrostomy tube placement.

## Introduction

The treatment of urolithiasis and endourology has been revolutionized with the advent of modern and minimally invasive techniques. Percutaneous nephrolithotomy (PCNL) represents an important advance that significantly reduces the number of lumbotomies performed for benign lithiasis [1]. The concept of percutaneous tubeless nephrolithotomy was to no longer put a nephrostomy tube at the end of the procedure and to drain the renal cavities only with a ureteral stent [2].

Through this work, our aim was to study the effectiveness, safety, and morbidity of tubeless PCNL by comparing it to the standard technique.

## Materials and methods

We retrospectively analyzed data from 125 patients who had undergone PCNL in our Urology Department between January 2015 and December 2017.

In our study, we included patients of both sexes over the age of 18 years in which the renal cavities were not perforated perioperatively. Patients with a single kidney or a congenital malformation and in whom more than one caliceal puncture was performed were excluded. No patient had a supracostal puncture or bilateral PCNL. All patients had a preoperative computed tomography urography (CTU) (a non-injected CT scan in patients with impaired renal function) and negative urine culture. All PCNLs were performed under general anesthesia in the Galadakao-modified supine Valdivia Uria position.

The patients were divided into two groups:

Group 1 (standard PCNL) (n = 74) in which the renal cavity drainage was provided by a nephrostomy tube (Fr 18) and a double-J ureteral stent or ureteral catheter.

Group 2 (tubeless PCNL) (n = 51): No nephrostomy was performed and a double-J catheter was kept in for one month.

PCNL results were considered good in the absence of residual lithiasis or less than 5 mm residual stones. We considered bleeding as a major complication when a blood transfusion was needed. We evaluated postoperative pain by the usage or non-usage of opioid analgesics.

Age, medical history, PCNL efficacy, specific and nonspecific complications, postoperative pain, and duration of hospitalization were compared between the two groups.

Statistical analysis

Average comparisons in two independent series were performed using Students T-test. Pearson's Chi-square test was used to compare percentages in independent series. The significance level was set at 0.05.

## Results

The clinical and demographic characteristics of our patients are detailed in Table 1. No statistically significant differences were noted between the two groups regarding age, sex, medical history, creatinine clearance, and prior surgery for lithiasis or shock wave lithotripsy (SWL).

**Table 1:** Patient Characteristics HTN: hypertension; PCNL: percutaneous nephrolithotomy; SWL: shock wave lithotripsy

	Standard PCNL	Tubeless PCNL	P-value
Age average (years)	53	50	0.32
Sex-ratio	1.39	1.42	0.94
Diabetes	24.3%	19.6%	0.53
HTN	32.4%	27.4%	0.55
Coronary disease	13.5%	9.8%	0.53
Kidney failure	9 (12.2%)	3 (5.9%)	0.24
History of SWL	20.3%	27.4%	0.35
History of surgery (for same-side lithiasis)	21.6%	19.6%	0.78

Regarding the treated stones (Table 2), it was found that there were more staghorn stones in the PCNL group with nephrostomy (p = 0.007) and more pelvicalyceal stones in the tubeless group (p = 0.037). Patients who had standard PCNL had larger stones (p = 0.008) with no statistically significant difference between the two groups regarding stone numbers (p = 0.14).

**Table 2:** Characteristics of Treated Stones PCNL: percutaneous nephrolithotomy

	Standard PCNL	Tubeless PCNL	p-value
Average number	1.5	1.8	0.14
Size	3.81 cm	3.16 cm	0.008
Hydronephrosis:			0.43
-Absent	24 (32.4%)	21 (41.2%)	
-Mild	34 (45.9%)	23 (45.1%)	
-Severe	16 (21.6%)	7 (13.7%)	
Stone site:			
-Pelvic	15 (20.3%)	12 (23.5%)	0.66
-Staghorn	35 (47.3%)	12 (23.5%)	0.007
-Pelvicalyceal	20 (27%)	23 (45.1%)	0.037
-Calyceal	4 (5.4%)	45 (7.8%)	0.58

No statistically significant difference was found between our two groups concerning the effectiveness of the PCNL (p = 0.13). Patients who had a tubeless PCNL had more postoperative infectious complications than the standard PCNL group (p = 0.042). No statistically significant differences were noted for other complications, postoperative pain (p = 0.51), or length of hospital stay (p = 0.16) (Table 3).

**Table 3:** Complication Rate, Length of Hospital Stay, and Degree of Postoperative Pain PCNL: percutaneous nephrolithotomy; PRBCs: packed red blood cells; SWL: shock wave lithotripsy

	Standard PCNL	Tubeless PCNL	p-value
Positive results	44 (59.5%)	37 (72.5%)	0.13
Complimentary SWL	22 (29.7%)	11 (21.6%)	0.31
Number of patients transfused	7 (9.5%)	3 (5.9%)	0.47
Number of patients transfused with 4 or more PRBCs	5 (6.8%)	2 (3.9%)	0.45
Pyelonephritis	2 (2.7%)	6 (11.7%)	0.042
Postoperative fever	0	2 (3.9%)	0.082
Urinary fistula	5 (6.8%)	0	0.058
Urinoma	1 (1.3%)	1 (2%)	0.79
Pleural breach	0	0	--
Digestive breach	1 (1.3%)	0	0.40
Arteriovenous fistula	0	1 (2%)	0.23
Non-specific complications	0	0	--
Postoperative pain (use of opioids)	3 (4%)	1 (2%)	0.51
Average length of hospital stay (days)	3.81	3.20	0.167

## Discussion

Tubeless PCNL was introduced by Bellman in 1997 which consisted of performing a PCNL without nephrostomy drainage at the end of the procedure [3-4].

A literature review confirms that this alternative is safe, effective, and reduces postoperative morbidity and length of hospital stay [2, 5-6], even in obese patients [7], after a supracostal puncture [8], and in patients presenting with large stones [9].

There is currently no consensus on the need for post-PCNL drainage, size and number of nephrostomy catheters (large or small, single or multiple), and type of ureteral drainage (ureteral catheter, double-J, mono-J, no drainage). This choice will often depend on the outcomes and difficulties encountered during the procedure and the surgeon's habits [10].

In our series, tubeless PCNL was performed in the absence of purulent retention, residual fragments, or significant bleeding. Good outcomes, as well as the rate of perioperative and postoperative complications, were similar to those of standard PCNL, apart from infectious complications which were more present in the standard PCNL group. Our results are consistent with those observed in almost all published series, except for the infectious complications [2, 5-6, 9].

However, unlike most authors, we did not find a statistically significant difference between the two techniques regarding postoperative pain and length of hospital stay.

According to a number of studies, whether or not a nephrostomy catheter was used depended essentially on the surgeon's choice, the outcome, and the difficulties encountered during the procedure (Table 4).

**Table 4:** A Sample of Published Series Comparing Standard PCNL Versus Tubeless

Study	Study type	Results	Nephrostomy placement criteria	Notes
Singh et al. (2008) [6]	Prospective randomized - Standard PCNL: 30 -Tubeless PCNL: 30	PCNL tubeless: Less length of stay and postop pain; Standard PCNL: Longer procedures with more X-ray exposition, urinary fistula, and hemorrhagic complications	Tubeless if stone < 3 cm	Standard PCNL: The procedure length and higher complication rates showcase the difficulties encountered in this group of patients.
Istanbulluogluet al. (2010) [11]	Retrospective: Standard PCNL: 92; Tubeless: 41; Totally tubeless: 43	Standard PCNL: Longer hospital stay, more opioids used in postop	Urine color	Selection bias: Tubeless was performed only in absence of hemorrhagic complications
Garofalo et al. (2013) [5]	Retrospective: Standard PCNL: 203; Tubeless PCNL: 114	PCNL Tubeless: Shorter hospital stay; Standard PCNL: More postop pain	Tubeless if no bleeding, no renal cavity perforation, and no significant residual lithiasis	These inclusion criteria could explain the advantages found in the tubeless group
Rifaioglu et al. (2014) [12]	Retrospective; Standard PCNL: 117; Tubeless: 107	Standard PCNL: Longer procedure, more hemorrhagic complications. PCNL tubeless: Better results	Surgeon's choice	The presence of residual calculi often motivated the placement of a nephrostomy which explains the higher stone-free rate in the tubeless group
Isac et al. (2014) [20]	Retrospective; Standard PCNL: 76; Tubeless: 83	PCNL Tubeless: Shorter hospital stay and less postop pain	Surgeon habits: Tubeless PCNL: No nephrostomy regardless of complications and residual fragments	More staghorn stones in the standard PCNL group. Less residual stones in the tubeless group which could be explained by 1) experienced surgeon, 2) easier stones to treat
Lu et al. (2012) [14]	Prospective randomized: 16 mini-PCNL (+ nephrostomy) versus 16 mini-PCNL tubeless	PCNL tubeless: Shorter hospital stay, less postoperative pain	2 randomized groups; (Among inclusion criteria: stone < 4 cm)	High stone-free rate: (Tubeless: 87.5%; Standard: 81.3%); Explained by: less operative difficulties, surgeon's experience, stone characteristics (Size < 4 cm, site not mentioned)
Zhao et al.(2016) [15]	Prospective randomized; 15 patients: PCNL with nephrostomy without ureteral stent; 15 patients: PCNL without nephrostomy with ureteral stent	Nephrostomy group: More postoperative comfort. No difference concerning complications, length of stay, and postoperative pain.	2 randomized groups; Tubeless: No residual fragments	Selection of patients: 100% stone-free. Patients felt more discomfort with ureteral stents than with a nephrostomy catheter
Jiang et al. (2017) [16]	Prospective randomized (90 patients): 30 PCNL (+ néphrostomy only); 30 PCNL (+ ureteral catheter only); and 30 PCNL (+ JJ stent only).	PCNL (with nephrostomy only): Longest hospital stay and more postop pain than other two groups; PCNL (+ JJ stent): More discomfort.	Randomized study; Selection criteria: stone < 2 cm, no bleeding, no residual calculi, no renal cavity perforation	Selection bias: Small stones, easier to treat - Fewer complications and perioperative difficulties.

PCNL without nephrostomy drainage is usually performed in the absence of residual fragments and perioperative complications, as well as in patients who are not considered at risk of complication [5, 11-13].

Several authors who studied tubeless PCNL included relatively easily treatable stones in regards to size, number, and site [11, 13-17].

Many authors compared the efficiency of conventional and tubeless PCNL based on the stone-free rate and the size of the residual fragments, whereas it seems to us that whether or not a nephrostomy catheter is placed at the end of the procedure has no direct relationship with the fragmentation and extraction of stones.

Regarding the length of hospital stay, which is shorter in the tubeless PCNL group according to the majority of authors [9, 13, 18], longer hospital stays in the standard PCNL group may be the consequence of restricted selection criteria within the tubeless group. There is no reason for nephrostomy catheter placement to delay the patient's discharge when its removal only takes a few minutes.

Postoperative pain has aroused the interest of most studies dealing with tubeless PCNL. It is agreed upon that the nephrostomy catheter is a source of postoperative pain [2-3, 17, 19]. However, is the pain caused directly by the catheter's irritation? Weren't the factors which led to the nephrostomy placement involved in the genesis of postoperative pain? Does the ureteral stent participate in the patient's discomfort as well [16]?

In prospective randomized studies, the choice to drain or not by a nephrostomy catheter by a surgeon whose tendency was to perform tubeless PCNL largely depended on the outcomes, difficulties, and complications encountered [20-23].

In fact, we believe that the slightest complication or procedural difficulty will motivate the surgeon to place a nephrostomy catheter in order to have a wider security margin. This selection bias may falsely confirm the results observed with the tubeless PCNL studies, which remain largely dependent on the surgeon's experience and habits, as well as the inclusion criteria of the published series.

In our opinion, selection bias exists in most tubeless PCNL studies (Table 4). We believe that nephrostomy drainage at the end of the procedure offers more security for the patient and reduces the risk of complications which can be sometimes fatal.

## Conclusions

The majority of authors agree that tubeless PCNL is a safe and effective technique and that the nephrostomy catheter is responsible for an increase of postoperative morbidity. According to the results of our series, the tubeless technique does not provide any advantage, especially regarding postoperative morbidity. A critical review of the literature shows that nephrostomy catheter can be a victim of selection bias.

## References

[1] Doré B (2006). Complications of percutaneous nephrolithotomy: risk factors and management (Article in French). Ann Urol (Paris).

[2] Saussine C, Lechevallier E, Traxer O (2008). Tubeless PCNL (Article in French). Prog Urol.

[3] Bellman GC, Davidoff R, Candela J, Gerspach J, Kurtz S, Stout L (1997). Tubeless percutaneous renal surgery. J Urol.

[4] Yoon GH, Bellman GC (2008). Tubeless percutaneous nephrolithotomy: a new standard in percutaneous renal surgery. J Endourol.

[5] Garofalo M, Pultrone CV, Schiavina R (2013). Tubeless procedure reduces hospitalization and pain after percutaneous nephrolithotomy: results of a multivariable analysis. Urolithiasis.

[6] Singh I, Singh A, Mittal G (2008). Tubeless percutaneous nephrolithotomy: is it really less morbid?. J Endourol.

[7] Yang RM, Bellman GC (2004). Tubeless percutaneous renal surgery in obese patients. Urology.

[8] Shah HN, Hedge SS, Shah JN, Bansal MB (2006). Safety and efficacy of supracostal access in tubeless percutaneous nephrolithotomy. J Endourol.

[9] Tefekli A, Altunrende F, Tepeler K, Tas A, Aydin S, Muslumanoglu AY (2007). Tubeless percutaneous nephrolithotomy in selected patients: a prospective randomized comparison. Int Urol Nephrol.

[10] Hoznek A, Rode J, Ouzaid I (2012). Modified supine percutaneous nephrolithotomy for large kidney and ureteral stones: technique and results. Eur Urol.

[11] Istanbulluoglu MO, Cicek T, Ozturk B, Gonen M, Ozkardes H (2010). Percutaneous nephrolithotomy: nephrostomy or tubeless or totally tubeless?. Urology.

[12] Rifaioglu MM, Onem K, Buldu I, Karatag T, Istanbulluoglu MO (2014). Tubeless percutaneous nephrolithotomy: yes but when? A multicentre retrospective cohort study. Urolithiasis.

[13] Desai MR, Kukreja RA, Desai MM, Mhaskar SS, Wani KA, Patel SH, Bapat SD (2004). A prospective randomized comparison of type of nephrostomy drainage following percutaneous nephrostolithotomy: large bore versus small bore versus tubeless. J Urol.

[14] Lu Y, Ping JG, Zhao XJ, Hu LK, Pu JX (2013). Randomized prospective trial of tubeless versus conventional minimally invasive percutaneous nephrolithotomy. World J Urol.

[15] Zhao PT, Hoenig DM, Smith AD, Okeke Z (2016). A randomized controlled comparison of nephrostomy drainage versus ureteral stent following percutaneous nephrolithotomy using the Wisconsin StoneQOL. J Endourol.

[16] Jiang H, Huang D, Yao S, Liu S (2017). Improving drainage after percutaneous nephrolithotomy based on health-related quality of life: a prospective randomized study. J Endourol.

[17] Lojanapiwat B, Soonthornphan S, Wudhikarn S (2001). Tubeless percutaneous nephrolithotomy in selected patients. J Endourol.

[18] Feng MI, Tamaddon K, Mikhail A, Kaptein JS, Bellman GC (2001). Prospective randomized study of various techniques of percutaneous nephrolithotomy. Urology.

[19] Gupta NP, Mishra S, Suryawanshi M, Seth A, Kumar R (2008). Comparison of standard with tubeless percutaneous nephrolithotomy. J Endourol.

[20] Isac W, Rizkala E, Liu X, Noble M, Monga M (2014). Tubeless percutaneous nephrolithotomy: outcomes with expanded indications. Int Braz J Urol.

[21] Crook TJ, Lockyer CR, Keoghane SR, Walmsley BH (2008). A randomized controlled trial of nephrostomy placement versus tubeless percutaneous nephrolithotomy. J Urol.

[22] Agrawal MS, Agrawal M, Gupta A, Bansal S, Yadav A, Goyal J (2008). A randomized comparison of tubeless and standard percutaneous nephrolithotomy. J Endourol.

[23] Bhat S, Lal J, Paul F (2017). A randomized controlled study comparing the standard, tubeless, and totally tubeless percutaneous nephrolithotomy procedures for renal stones from a tertiary care hospital. Indian J Urol.

